# The myodural bridge existing in the *Nephocaena phocaenoides*

**DOI:** 10.1371/journal.pone.0173630

**Published:** 2017-03-09

**Authors:** Pei Liu, Chan Li, Nan Zheng, Qiang Xu, Sheng-Bo Yu, Hong-Jin Sui

**Affiliations:** 1 The First Affiliated Hospital of Dalian Medical University, Dalian, China; 2 Department of Anatomy, College of Basic Medicine, Dalian Medical University, Dalian, China; 3 Department of Radiology, The 403 Affiliated Hospital of Chinese PLA General Hospital, Dalian, China; 4 Dalian Hoffen Preservation Institution, Dalian, China; Universita degli Studi di Bari Aldo Moro, ITALY

## Abstract

Recent studies have identified that the myodural bridge (MDB) between the rectus capitis posterior minor (RCPmi) and the cervical spinal dura mater in the posterior atlanto-occipital interspace in humans. And it was supposed that the MDB may play essential physiological roles. As a result, the MDB is possibly a highly conserved structure in the evolution of mammals. However, there is little confirmative description about the existence of the MDB in marine mammals. The objective of this study was to explore the existence and the fiber property of the MDB in the *Neophocaena phocaenoides*. Six cadavers of the *Neophocaena phocaenoides* with formalin fixation were used in this study. One was used for head and neck CT scanning and three-dimensional (3D) reconstruction and suboccipital region dissection, two were for sectional observation by P45 plastinated sheets of head and neck, and three were for histological analysis of suboccipial structures. This is the first study to demonstrate the existence of the MDB in the aquatic mammals. The rectus capitis dorsal minor (RCDmi) originated from the inferior border of the occiput and inserted into the cervical spinal dura mater. At the ventral aspect of the RCDmi, the MDB directly extended through the posterior atlanto-occipital interspace and connected with the cervical spinal dura mater which was consisted of type Ⅰ collagen. In addition, the dorsal atlanto-occipital membrane was not found in the *Neophocaena phocaenoides*. The tendinous myodural bridge extended from the RCDmi to the spinal dura mater through the posterior atlanto-occipital interspace in the *Neophocaena phocaenoides*.

## Introduction

The existence of a bundle of connective tissue between the suboccipital musculature and cervical spinal dura mater in the atlanto-occipital interspace in humans has been generally accepted in recent years [1]. This kind of anatomical connection was termed the myodural bridge (MDB) according to previous studies [2, 3]. The MDB of the rectus capitis posterior minor (RCPmi) was firstly reported in humans atlanto-occipital interspace by Khan [2] and Hack [3]. Their findings dissected the formation of the human MDB. The RCPmi gave off dense connective tissue connecting with the posterior atlanto-occipital membrane (PAOM), which merged with the cervical spinal dura mater, forming the “posterior atlanto-occipital membrane-spinal dural complex” (PAOM-SDC). The MDB between the RCPmi and cervical spinal dura mater was re-confirmed via anatomical dissection, MRI scanning, sheet plastination, and/or histological observation of human cadaveric specimens by studies of Mitchell [4], Humphreys [5], Nash [6] et al. Furthermore, the MDB was found to be constituted by tendon-like, muscle-like, or fascia-like tissue, and the main type of fiber was tendon-like [6, 7]. It was further verified that the RCPmi directly gave off a tendinous subdivision and connected with the PAOM according to P45 plastination sheets observation [6, 8]. Based on structural features of the MDB, it was supposed that the MDB may play essential physiological roles in cerebrospinal fluid (CSF) circulation [8–11], tension-sensation of cervical dura mater and/or cervical dura mater position-keeping [1–13]. Consequently, the MDB may be a highly conserved structure in the evolution of terrestrial mammals such as humans. However, the confirmative information about the existence of the MDB in marine mammals remained scant.

The *Neophocaena phocaenoides* is a small, toothed whale that belongs to the mammalian order of Cetacea. Commonly seen in the shallow coastal water of torrid zone to warm-temperate-zone around east Asia. Unlike humans, the first three cervical vertebrae are fused as one bone, and the atlanto-occipital joint was actually formed by occiput and the fused vertebrae [14–16]. As a result, the *Neophocaena phocaenoides* moves its head much less flexibly than humans. We initiated this study to explore the existence and the fiber property of the myodural bridge of the *Neophocaena phocaenoides*.

### Ethics statement

Six *Neophocaena phocaenoides* cadavers were collected successively from beaches in Dalian with the permission of Chinese Authorities for Animal Protection. All *Neophocaena phocaenoides* died naturally. The cadavers of these animals were permitted for scientific research under the approval of the Ethics Committee of Dalian Medical University. The obtained specimens were embalmed through the aorta with 10% formalin solution.

## Methods

### CT three-dimensional reconstruction

Head and neck of one specimen was continuously scanned using GE Amira 128-row VCT, and dual phase serial computer tomography (CT) images were obtained, with a slice thickness and pitch set to 0.6mm. The CT was set to 120 kv and 140 mAs to produce an image matrix of 512×512 pixels and a field of view of 230mm. All CT images were obtained in Digital Imaging and Communications in Medicine (DICOM) format for the purpose of reconstruction. According to the particularities of anatomy structures and traits of images, Visualization software Mimics 10.01 (Materialise, Leuven, Belgium) was used for image modeling and reconstruction. The 2-dimensional (2D) slices of both sides of the skeleton DICOM files were automatically combined and converted to 3D models.

### Dissection of suboccipital region

After CT scanning, a layer-by-layer dissection of posterior occipital region was performed to expose the atlanto-occipital interspac. An incision was made along the dorsal midline of the neck, the skin and fat were removed until the superficial posterior occipital muscles were exposed. Subsequently, the posterior occipital muscles, including were identified respectively and removed carefully until all the deep suboccipital muscles could be seen. Following that, the suboccipital muscles were detached from their cranial attachment respectively to reveal the ventral aspect of the muscles and as well as the structural connections between them. RCDma was detached from its cranial attachment and was lifted aside untill the RCDmi was exposed. The RCDma, the RCDmi, and the deep structures underneath the RDCmi in the atlanto-occipital interspace were isolated by an electric hand saw and preserved for P45 sheet plastination and histology.

### P45 sheet plastination

Two *Neophocaena phocaenoides* specimens were sliced sagittally for P45 sheet plastination. Anatomical structures of the posterior occipital region and the connections between the suboccipital muscles and the cervical dura mater were observed according to P45 sheet plastination. The experimental procedure is described below [17]:

#### Slicing

The embalmed specimens of the head and neck were frozen at -70℃ for two weeks and then embedded in polyurethane foam and frozen at -70℃ again for two days. After freezing, 3 mm sagittal slices were made from side to side with a high-speed band saw.

#### Bleaching

All the slices were rinsed overnight in cold running water, and afterwards, the slices were immersed in 5% dioxogen overnight.

#### Dehydration

After bleaching, the slices were dehydrated with 100% acetone by the freeze substitution method.

#### Casting and forced impregnation

After dehydration, the casting mold was prepared. The slices were lifted from the acetone bath and placed between two glass plates. The molds were then filled with polyester (Hoffen polyester P45, Dalian Hoffen Bio-Technique Co. Ltd., Dalian, P. R. China.).

The filled mold was placed upright into a vacuum chamber at room temperature for impregnation. The absolute pressure was slowly decreased to 20, 10, 5, and 0 mm Hg, according to the rate of bubble releasing. The pressure was maintained at 0 mm Hg until bubbling ceased. The impregnation time lasted for more than eight hours.

#### Curing

After the vacuum was released, the air bubbles within the sheets were checked and removed. The top of the mold was clamped with large fold back clamps, and the sheet was then ready for curing. The sheets were cured using a heated water bath and were placed upright in the water bath at 40℃ for 3 days. After curing, the sheets were removed from the bath and cooled to room temperature in a rack. The slices were then removed from the flat chamber and covered appropriately with adhesive plastic wrap for protection. The head-neck sheets were then observed and photographed.

### Histology

Three embalmed cadavers of the *Neophocaena phocaenoides* were dissected along the median sagittal plane to isolate a unit including the suboccipital musculature, outer periosteum of the occiput, and the perosteum of the fused cervical vertebrae. In order to maintain the integrity of the MDB with both the RCDmi and the spinal dura mater, the spinal dura mater and spinal cord were removed from the bodies with the isolated unit. 2×2 cm sections were dissected for multiple histological stainings.

All sections were dehydrated with ethanol and xylene and infiltrated with melted paraffin wax. Then the sections were embedded in the filled mold and shaped when it cooled to room temperature. A rotary microtome was used to make 6-μm-thick sections, and the glass microscope slides were prepared for rehydration and staining with hematoxylin and eosin (HE), Van Gieson (VG, picric acid and acid fuchsin) staining and Sirius Red (SR) in saturated carbazotic acid (see S1 File for detailed staining methods).

Disposed samples were put in glass coverslips after stain. HE staining and VG staining sections were observed under a light microscope. Images were photographed and analyzed by NIS imagine. The results of SR in saturated carbazotic acid staining was acquired both through light microscope and polarizing microscope.

## Results

### A wide dorsal atlanto-occipital interspace was found between the fused cervical vertebrae and occiput based on 3D reconstruction

**Fig 1:**
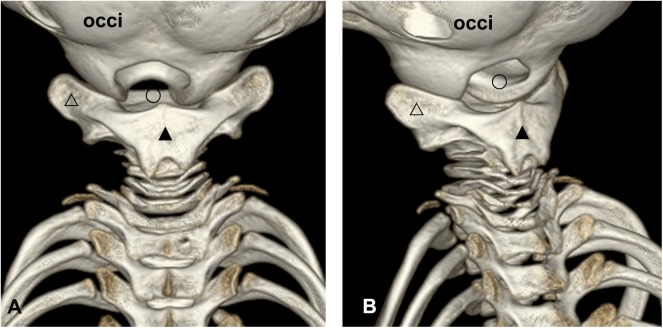
3D-reconstruction of cranium and cervical vertebrae of the Neophocaena phocaenoides. A: dorsal view of the suboccipital region. B: left lateral aspect of the suboccipital region. occi: occiput. △: the fused transverse processe of the first three cervical vertebrae. ▲: the fused spinous processe of the first three cervical vertebrae. ○: the atlanto-occipital interspace.

It was demonstrated that there was a broad interspace between the fused cervical vertebrae and occiput. The occipital condyles protruded caudally to a remarkable degree and formed the lateral borders of the atlanto-occipital interspace. The first three cervical vertebrae fused into one bone and formed the caudal border of the interspace, shaping the atlanto-occipital interspace into an inverted “U” as a result. At the dorsal aspect, the fused spinous processes formed a broad dorsal plate located almost horizontally (marked as “▲”, Fig 1). The transverse processes of the first three cervical vertebrae were fused into a solid unit as well (marked as “△”, Fig 1). This fused transverse process projected bilaterally, whose anterior aspect had a concave spherical articular surface to join with the occipital condyles (Fig 1A). In the lateral aspect of the suboccipital region, the fused transverse processes could be seen as well. For the remaining four vertebrae, bodies and arches were relatively smaller than the first three. The intervertebral interspace was narrower between adjacent vertebrae, compared with the atlanto-occipital interspace (Fig 1B).

### Three posterior occipital muscles in posterior occipital region were identified according to gross anatomy

**Fig 2:**
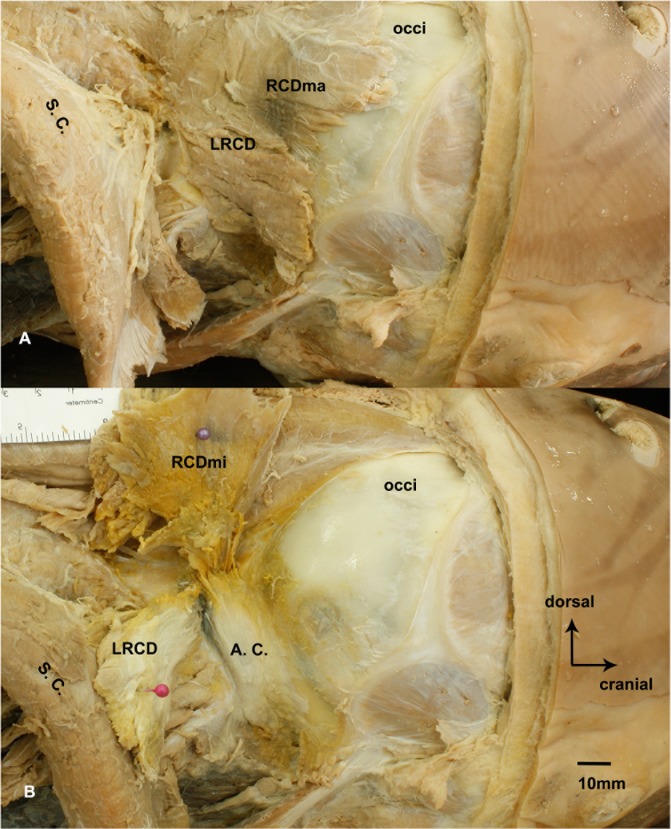
The suboccipital muscles of the *Neophocaena phocaenoides* in the atlanto-occipital interspace. A: the superficial layer of suboccipital musculature. B: the deep layer of suboccipital musculature. A. C.: articular capsule of the atlanto-occipital joint. LRCD: the lateral rectus capitis dorsal. RCDmi: the rectus capitis dorsal minor. RCDma: the rectus capitis dorsal major. S. C.: semispinalis capitis. occi: occiput.

For gross anatomy, in the deep suboccipital region, only three muscles were found. According to their courses and location, they were termed the lateral rectus capitis dorsal (LRCD), the rectus capitis dorsal major (RCDma) and the rectus capitis dorsal minor (RCDmi), respectively (Fig 2). The LRCD was found bilaterally along the lateral borders of the atlanto-occipital interspace, attached to occiput and the transverse process of the fused cervical vertebrae (Fig 2A). The RCDma attached to occiput and the transverse process, and extended middorsally into the atlanto-occipital interspace (Fig 2B). The RCDmi was found deep to the RCDma, when we cut off the cranial attachment of the RCDma and lifted aside the muscle, the RCDmi was observed originating from occiput and extending into the atlanto-occipital interspace.

Surprisingly, the oblique capitis anterior (OCA), the oblique capitis posterior (OCP), or the dorsal atlanto-occipital membrane (DAOM) were not found in the suboccipital region.

### The RCDmi directly connected with the cervical spinal dura mater based on P45 plastinated sheets

**Fig 3:**
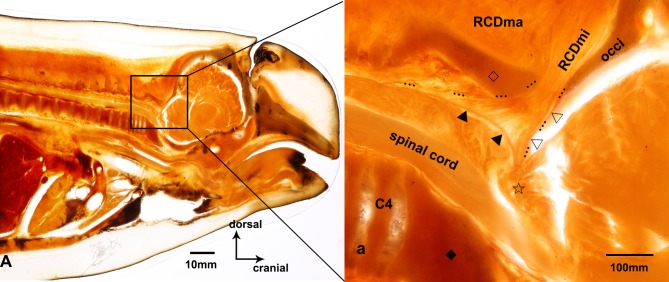
Sagittal sectional P45 plastinated sheet of suboccipital region of the *Neophocaena phocaenoides*. A: Sagittal section of P45 plastinated sheet of the *Neophocaena phocaenoides*. a: The magnified area of suboccipital region. RCDmi: the rectus capitis dorsal minor. RCDma: the rectus capitis dorsal major. C4: the fourth cervical vertebra. occi: occiput. ☆: cerebellomedullary cistern. ▲: spinal dura mater. △: cerebral dura mater. ◇: the fused spinous process of the first three cervical vertebrae. ◆: the fused vertebral body of the first three cervical vertebrae. …: the myodural bridge.

The relationship between the bones and muscles was clearly visible on the P45 plastinated sheets of the atlanto-occipital interspace (Fig 3). Throughout the sheet, all of the muscular fibers of the RCDmi extended directly into the atlanto-occipital interspace, terminated at the cervical spinal dura mater, where a reverse angle of the cranial and spinal dura mater was observed. Additionally, no posterior atlanto-occipital membrane was found in the atlanto-occipital interspace.

### The RCDmi gave off its tendon and connected intensively with the cervical spinal dura mater based on histological staining

**Fig 4:**
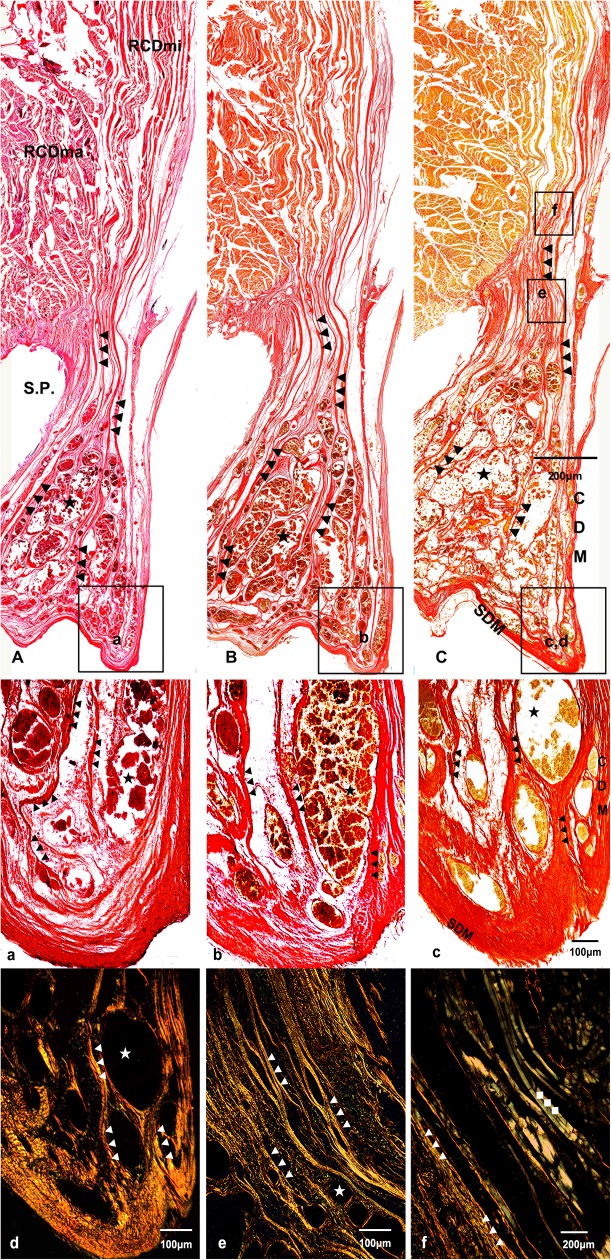
Sagittal sectional histological sheet of suboccipital region of the *Neophocaena phocaenoides*. A, a: results of Hematoxylin and eosin staining. B, b: results of VG (picric acid and acid fuchsin) staining. C, c, d, e, f: results of Sirius Red in saturated cabazotic acid staining. A, B, C, a, b, c were under 4× light microscope. d and e were under 5× polarized microscope, and f was under 10× polarized microscope. RCDmi: the rectus capitis dorsal minor. RCDma: the rectus capitis dorsal major. SDM: spinal dura mater. CDM: cerebral dura mater. S. P.: fused spinous process of the first three vertebrae. ▲: the myodural bridge. ★: venous plexus. ◆: the muscle fibers of the RCDmi.

During histological analysis, the RCDma, the RCDmi and the MDB were identified definitively based on the results of HE, VG and SR in saturated carbazotic acid staining. The results of HE staining revealed muscular fibers of the RCDma were between occiput and the fused cervical vertebrae. The muscular fibers of the RCDmi arranged in parallel with each other, and entered the epidural space through the atlanto-occipital interspace, finally terminating at the cervical spinal dura mater. In regard to dura mater, the cranial dura mater came out of the foramen magnum and reversed sharply to transform into the cervical spinal dura mater, where the dense connective fibers of RCDmi terminated. (Fig 4A)

The result of VG staining showed that muscle fibers of the RCDmi were stained in yellow, while the dense connective fibers, originating from the RCDmi, were stained in red, indicating that these connective fibers were collagenous fibers (Fig 4B). Furthermore, for SR in saturated carbazotic acid staining, these collagenous fibers were red under light microscope (marked as “▲”, Fig 4C, c) and highlighted in yellow under polarized microscope (marked as “▲”, Fig 4D, 4E and 4F), indicating the connections were composed of collgen Ⅰ. Besides, the muscle fibers of the RCDmi were highlighted in green under polarized microscope (marked as “◆”, Fig 4F), indicating the muscle fibers were composed of collgen Ⅲ. Since collagen Ⅰ is the major component of tendons, the dense connective fibers of the RCDmi were tendinous.

Additionally, a large number of venous plexus filling in the epidural space was found around the cervical spinal cord in *Neophocaena phocaenoides*.

## Discussion

*Neophocaena phocaenoides* is a small, toothed whale which belongs to mammalian order of Cetacea, lives in both fresh and marine coastal waters. Unlike humans and other terrestrial mammals, its first three cervical vertebrae are fused, so the movement of head and neck tends not to be as movable and polydirectional. *Neophocaena phocaenoides* therefore could be a good example to study whether the MDB is a highly conserved structure in the evolution of mammals and to explore the physiological functions of MDB.

This is the first study to demonstrat the existence of the MDB in the *Neophocaena phocaenoides*. Based on the morphological observations, it was observed that the RCDmi originated from the occiput, muscle bundles from the RCDmi lined vertically to the dura mater and travlelled through the atlanto-occipital interspace. After entering the epidural space, all tendinous bundles of the RCDmi terminated at the posterior surface of cervical spinal dura mater. In summary, this study found that the RCDmi was a single and specialized origin for the MDB. Furthermore, this study revealed that the fibers of the MDB were composed of collagen I.

Unlike humans, the muscles in the suboccipital region of the *Neophocaena phocaenoides* did not form the suboccipital triangle [1, 11]. Unlike humans, neither the OCA nor the OCP in the *Neophocaena phocaenoides* was observed, to our surprise. Only three muscles in parallel with each other could be seen, which were the LRCD, the RCDma and the RCDmi. The muscle fibers of the RCDmi projected into theatlanto-occipital interspace. Additionally, no DAOM was found in the *Neophocaena phocaenoides*. These results collectively indicate that the MDB of the *Neophocaena phocaenoides* is different from humans: in humans, the PAOM connects the occiput and the atlas, and is mainly composed of tendon fibers, the fascia of the RCPmi and the perivascular sheathes of the vertebral vessels [6].

It may be possible that the direct connection of the RCDmi of the *Neophocaena phocaenoides* to the cervical dura mater could result in stronger effects across the MDB. We explored the fiber type histology, as the fiber type affects the tension of a particular tissue. According to our research, the MDB of the *Neophocaena phocaenoides* was tendinous in nature, and its fiber type was collagen I. Structures made up of collagen I tend to be highly resistant to strong tensile force [18–20].

During evolution, morphology of a body structure is influenced by its function as well as the living environment. The specialized morphologic features and fiber property of the MDB may play significant physiological roles in mammals. Such as preventing dura mater enfolding, stabilizing the spinal cord [1–13], and modulating the circulation of CSF [8–11, 21]. It is reasonable to suggest that flow rate and pressure of CSF could effectively affect CSF ciculation at the communication of the cranial cavity with the vertebral canal as the RCDmi contracts or relaxes. the tense MDB may transmit forces to spinal dura mater and may change the volume of the subdural space and modulate CSF pressure in the subarachnoid space [8–11, 21]. In humans, it is commonly known that the epidural space around the cervical spinal cord is filled with venous plexus [22–25]. Here in *Neophocaena phocaenoides*, we found a large amount of epidural venous plexus in the suboccipital region, the volume of these venous plexus could be changed continuously while the animal is swimming, due to the movements of the suboccipital muscles especially the RCDmi. For dolphins, the heart rate could reach to about 120 per minute on the surface of water, however, it goes down to 30–40 per minute underwater [14, 16, 26]. A previous research demonstrated that harbor porpoise (Phocoena phocoena) showed a mean heart rate around 125 per minute on the surface of water [24]. Although there is no data regarding to the diving heart rate of harbor porpoise, the finless porpoise and dolphins share the similar surfacing heart rate, and they may share the similar heart rate underwater. Since the pulsations of the arteries and veins are weak and slow underwater, the movements of suboccipital muscles could be considered as an significant power source of CSF circulation. Qiang Xu et al. scaned the atlanto-occipital interspace of 40 volunteers by MRI when their head rotated, and they found that the flow rate and volume of CSF were changed, possibly involving the MDB [21].

This study confirmed the existence of the MDB of the *Neophocaena phocaenoides* and analyzed its fiber property, and inspired us to speculate upon the particular physiological function of the MDB. Other mammalian orders need to be studied to supplement existing information on how broadly conserved the MDB is in mammals.

## Supporting information

S1 File Staining methods. (DOCX)
