# Supplementary material for: The myodural bridge existing in the Nephocaena phocaenoides
Source: PLoS One. 2017 Mar 9;12(3):e0173630. doi: 10.1371/journal.pone.0173630 (PMC5344499; doi:10.1371/journal.pone.0173630)
Supplement: S1 File — (DOCX) [file pone.0173630.s001.docx]

**S1 File. Staining methods**

**The H&E staining**

1 regular dewax and rehydrate

2 dye in hematoxylin (10 mins)

3 1% hydrochloric acid alcohol (1 min)

4 wash by running tap water

5 wash by DW briefly

6 dye in Eosin for 30 seconds

7 regular dehydration

8 clear by 2 changes xylene (5 mins each)

9 mount with neutral gum

**The Van Gieson staining**

1 regular dewax and rehydrate

2 dye in hematoxylin (10 mins)

3 1% hydrochloric acid alcohol (1 min)

4 wash by running tap water

5 wash by DW briefly

6 0.1% acid fuchsin solution (1X volume 1% acid fuchsin in 9X volume saturated carbazotic acid, dye for 1.5 mins)

7 regular dehydration

8 clear by 2 changes xylene (5 mins each)

9 mount with neutral gum

**The Sirius Red staining**

1 regular dewax and rehydrate

2 celestine blue solution：celestine B 1.25g, halotrichite 1.25g, DW 250ml. Boil to solve, filter after cooling down, add 30ml glycerin, and add concentrated sulfuric acid 0.5ml. Dye for 10 mins

3 wash by dw (3 times, 1 minute each)

4 0.1% sirius red solution (1X volume 1% sirius red in 9X volume saturated carbazotic acid, dye for 1.5 hours)

5 wash by running tap water (5 mins)

6 redye by hematoxylin (10 mins)

7 wash by running tap water (5 mins)

8 regular dehydration

9 clear by 2 changes xylene (5 mins each)

10 mount with neutral gum

DW=distilled water
